# C-reactive protein–albumin–lymphocyte (CALLY) index as a novel inflammatory marker for sarcopenia: evidence from a cross-sectional study

**DOI:** 10.55730/1300-0144.6363

**Published:** 2026-08-06

**Authors:** Yasemin POLAT ÖZER, Merve GÜNER, Serdar CEYLAN, Engin ÇEŞMECİ, Cafer BALCI, Burcu Balam DOĞU, Meltem Gülhan HALİL, Mustafa CANKURTARAN, Mert EŞME

**Affiliations:** 1 Division of Geriatrics, Department of Internal Medicine, Faculty of Medicine, Hacettepe University, Ankara, Turkiye; 2 Division of Geriatrics, Department of Internal Medicine, Faculty of Medicine, İstinye University, İstanbul, Turkiye; 3 Division of Geriatrics, Department of Internal Medicine, Antalya City Hospital, Antalya, Turkiye

**Keywords:** C-reactive protein-albumin-lymphocyte index, comprehensive geriatric assessment, inflammaging, older adults, probable sarcopenia

## Abstract

**Background/aim:**

Sarcopenia is a geriatric syndrome characterized by progressive impairments in skeletal muscle mass and performance, with chronic low-grade systemic inflammation playing a pivotal role in its development. The C-reactive protein–albumin–lymphocyte (CALLY) index reflects inflammatory burden alongside immune and nutritional status; however, its association with sarcopenia in older populations has yet to be sufficiently elucidated. The present study investigates the relationship between CALLY index values and probable sarcopenia (PS) in a large cohort of geriatric outpatients.

**Materials and methods:**

Included in this retrospective cross-sectional study were 2042 geriatric outpatients aged ≥65 years who were assessed between May 2022 and March 2025. PS was defined based on EWGSOP-2 criteria (handgrip strength or chair stand test), while sarcopenia risk was assessed using the SARC-F questionnaire (assessing strength, assistance with walking, rise from a chair, climb stairs, and falls). The CALLY index was calculated based on comprehensive geriatric assessment results and laboratory parameters. A complete case analysis was performed and patients with incomplete data were excluded. Group comparisons, logistic regression, ROC curve analyses, and correlation analyses were performed.

**Results:**

Of the participants, 1092 (53.5%) were classified as PS and 950 (46.5%) as normal. Lower CALLY index values were observed in those with PS and high SARC-F scores (p <0.001), and were independently associated with both PS and sarcopenia risk in logistic regression analysis. In addition, the CALLY index was negatively correlated with SARC-F, timed up and go, chair stand test, and 4-meter walking time, and positively correlated with handgrip strength (all p <0.001).

**Conclusion:**

Lower CALLY values were independently associated with both PS and sarcopenia risk among older adults, supporting its use as a practical and accessible biomarker alongside routine geriatric assessment. Due to the cross-sectional nature of the study, further prospective studies involving a larger cohort are necessary to verify these associations and to assess prognostic utility.

## 1. Introduction

Inflammaging, defined as a persistent low-grade inflammation that accompanies aging, has emerged as a key biological process underlying multiple age-associated disorders [1]. This inflammatory milieu in older adults contributes substantially to the development of sarcopenia, frailty, and other geriatric syndromes by hastening functional and physiological deterioration [2]. Sarcopenia is a condition that develops with age and is characterized by a gradual loss of skeletal muscle mass and strength that predisposes older adults to such unfavorable outcomes as reduced mobility, greater illness burden, and an elevated risk of morbidity and mortality [3]. Globally, sarcopenia affects 10%–27% of all older adults, with prevalence rates differing considerably across studies due to variations in diagnostic definition. The rate rises dramatically with advancing age, reaching nearly 50% in those aged ≥80 years [4, 5]. Although the etiology of sarcopenia is complex, chronic low-grade systemic inflammation is widely recognized as a significant factor in its pathogenesis.

Inflammaging is thought to accelerate muscle loss due to the activity of catabolic cytokines, as shown by higher levels of interleukin-6 (IL-6), tumor necrosis factor-α (TNF-α), and C-reactive protein (CRP) in affected individuals. [6–8]. The number of measurable indices of inflammation is increasing [9]. In the past decade there has been a growing body of research highlighting associations between sarcopenia, increased inflammation-based indices, and reductions in nutritional and immune status among older or vulnerable populations. Evidence from observational studies of both community-dwelling older adults and hospitalized patients indicates that inflammation-based indices reflecting inflammatory burden are frequently linked to reduced muscle mass and strength, along with a higher prevalence of sarcopenia [10–15]. However, how accurate these biomarkers are in predicting sarcopenia remains unknown. Among the available indices, the CRP–albumin–lymphocyte (CALLY) index has been proposed as a composite biomarker that reflects the interplay among inflammatory activity, immune function, and nutritional status [16].

The CALLY index functions as an innovative composite parameter, with several studies to date reporting its ability to predict 5-year survival and mortality in multiple malignancies. No defined cutoff value for the index exists, and various methods have been applied in the literature to define a value. Most notable among these approaches is the receiver operating characteristic (ROC) curve analysis [17–21], in which lower scores have been associated with greater inflammatory burden and a higher risk of mortality [19, 20, 22].

It is necessary to screen older patients for sarcopenia, to take the necessary precautions through early diagnosis and, if needed, to start treatment. Higher CALLY index levels are independently linked to a lower risk of sarcopenia in hospitalized patients, being a significant geriatric syndrome associated with negative health outcomes [23]. Reliable biomarkers are essential for the early diagnosis and risk assessment of sarcopenia; and recognizing this, the present study evaluates the association between the CALLY index and sarcopenia in older adults.

## 2. Materials and methods

### 2.1. Study design

**Figure 1:**
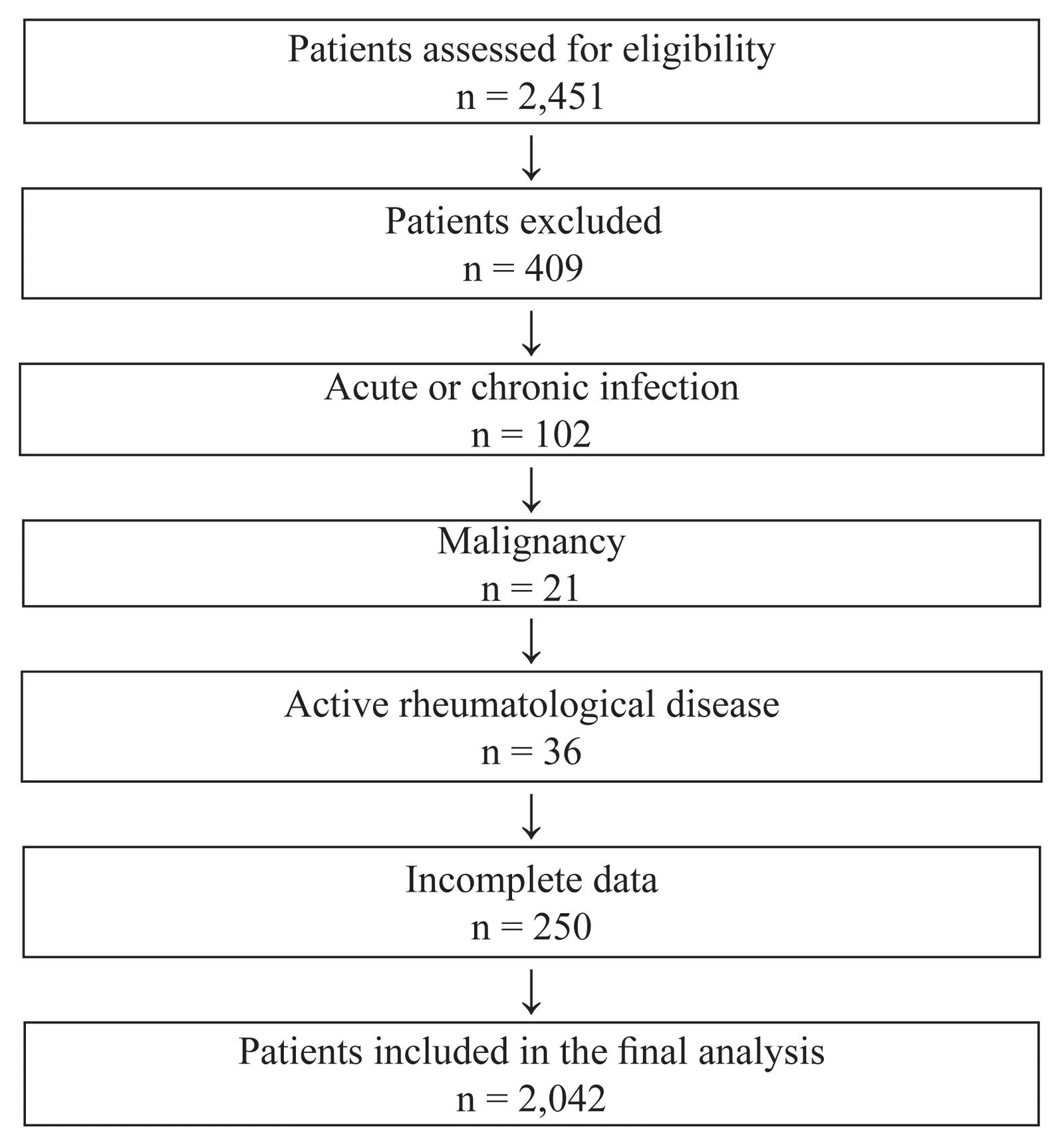
Flow diagram of the patient selection process.

This retrospective study was conducted in the Geriatrics department of our university hospital, in which a total of 2042 geriatric outpatients were assessed between May 2022 and March 2025. The patients were classified as either normal or probable sarcopenia (PS) according to the EWGSOP-2 criteria, based on the results of handgrip strength (HGS) and chair stand test (CST) results. Patients aged ≥65 who visited the outpatient clinic and met the study inclusion criteria were recruited and subjected to a comprehensive geriatric assessment (CGA), and those with acute or chronic infections, malignancies, active rheumatological diseases, or incomplete data were subsequently excluded from the study. The patient selection process is illustrated in Figure 1. A total of 2451 patients were initially evaluated for eligibility, and after excluding those with acute or chronic infections (n = 102), malignancies (n = 21), active rheumatological diseases (n = 36), and incomplete data (n = 250), 2042 patients with complete datasets were included in the final analysis.

### 2.2. Comprehensive geriatric assessment

CGA was performed as routine during outpatient clinic examinations and the results were recorded retrospectively using standardized tools. Functionality was assessed using the Katz Index of Activities of Daily Living (ADL), which assesses patients on a six-point scale based on their capacity to carry out basic care and daily activities, with lower scores indicating greater dependence. The patients’ ability to manage their personal shopping, meal preparation, housekeeping, transportation, and medication requirements was assessed using the Lawton–Brody Instrumental Activities of Daily Living (IADL) scale, in which assessments are made on a scale of 0–8, with lower scores signifying greater dependence [24, 25]. Cognitive performance was evaluated using the Standardized Mini-Mental State Examination (SMMSE), in which scores of ≤24 out of the maximum of 30 measuring orientation, memory performance, attentional capacity, numerical processing, recall, language, and motor skills are considered indicative of cognitive impairment [26, 27]. The Yesavage Geriatric Depression Scale (GDS) was utilized to screen for depression, and patients scoring >5 were scheduled for clinical depression assessment [28]. Nutritional status was determined using the Mini-Nutritional Assessment Short-Form (MNA-SF), in which scores of >11 indicate normal nutritional condition, scores of 8–11 indicate potential risk of malnutrition, and scores of ≤7 indicate malnutrition [29, 30]. Finally, frailty was assessed using the Clinical Frailty Scale (CFS), in which scores of ≥4 are indicative of frailty [31, 32].

### 2.3. CALLY index assessment

After overnight fasting, all participants underwent biochemical and complete blood count tests on the same day as their sarcopenia status assessment, with values calculated from the results of routine blood tests performed in the outpatient clinic. The CALLY index was calculated using the formula originally described by Iida et al. [17]: (Albumin (g/L) × Lymphocyte (k/mm^3^)) / (CRP (mg/L) × 10^4^)

### 2.4. Diagnosis of sarcopenia risk and probable sarcopenia

The patients were classified as either normal or probable sarcopenia (PS) according to the EWGSOP-2 criteria, based on HGS and CST results. The CST was performed with participants rising from a standard chair five times as quickly as possible without using their arms. HGS was assessed using a Takei handgrip dynamometer (Niigata City, Japan) for which the participants performed three grip attempts with their dominant hand, with a 1-minute rest between attempts. The highest measurement was noted for analysis, in accordance with EWGSOP-2 guidelines [33]. Physical performance was evaluated using the Timed Up and Go (TUG) test, which assesses the time taken for the participant to rise from a chair, traverse a distance of 3 meters, execute a turn, return to the chair, and sit. The participant’s 4-meter gait speed is also evaluated, measured over a 4 m track. Each assessment was conducted twice, and the mean of the two was used for analysis [34]. A gait speed of <0.8 m/s indicated slow walking, and a TUG time of ≥20 seconds was used as the cutoff for poor physical performance [33].

SARC-F is used to identify sarcopenia risk, a five-item questionnaire assessing strength, assistance with walking, rise from a chair, climb stairs, and falls. Each item is scored on a scale of 0–2, yielding a total maximum of 10; scores of ≥4 suggest potential sarcopenia risk [35, 36]. PS was considered present in participants with low muscle strength, indicated either by a low HGS score according to sex-specific reference thresholds (men <27 kg; women <16 kg) or by CST duration of at least 15 seconds [33].

### 2.5. Statistical analysis

Statistical analyses were performed using IBM SPSS Statistics, version 22.0 (IBM Corp., Armonk, NY, USA). The distribution of variables was analyzed using graphical tools (histograms and probability plots) in conjunction with the Kolmogorov–Smirnov test. Normally distributed data were expressed as mean ± standard deviation, whereas nonnormally distributed data were presented as median (interquartile range). Categorical variables were presented as numbers and percentages, and group comparisons were performed using the chi-square test or Fisher’s exact test, as applicable.

For continuous variables, the independent-samples t-test was used when parametric assumptions were met, and the Mann–Whitney U test otherwise. Correlation analyses were carried out using either Pearson or Spearman coefficients, depending on the data characteristics. The discriminatory performance of the CALLY was assessed with a ROC curve analysis, for which the optimal threshold was determined according to the Youden index. Sensitivity, specificity, positive predictive value, and negative predictive values were subsequently calculated.

Binary logistic regression analysis (enter method) was used to adjust for potential confounders. Variables that were statistically significant in the univariate analyses were included in multivariable logistic regression models. Multicollinearity among the candidate variables was assessed, and collinear variables were not included in the same model. The Hosmer–Lemeshow goodness-of-fit test was used to assess the model’s fit. No formal internal validation was performed.

A complete case analysis was performed, and patients with incomplete CGA data or missing laboratory parameters required for the CALLY index calculation (albumin, CRP, lymphocyte count) were excluded from the analysis. p-values below 0.05 were regarded as statistically significant. Ethical approval for the study was obtained from the institutional review board of the university hospital (date: 18.03.2025, protocol number SBA 25/241).

## 3. Results

The final study sample included 2042 patients, of whom 1335 (65%) were female and 707 (35%) were male with a median age of 73.0 [69–78]. Overall, 950 patients (46.5%) were classified as PS. Overall, 480 patients (23.5%) had high SARC-F scores (based on a cutoff value of 4). The sample was divided into two groups, as those with PS (n = 950, 46.5%) and those with normal status (n = 950, 46.5%) based on the EWGSOP-2 criteria. The patients’ sociodemographic features and comorbidities are summarized in Table 1 and the CGA parameters in Table 2. Comorbidities and geriatric syndromes were recorded for each participant and expressed as numbers and percentages (n, %). Those classified as PS were significantly older, with a median age of 75 (71–80) years, compared to 71 (68–75) years in the normal group (p <0.001). Statistically significantly higher rates of comorbidities were noted in the PS group, including hypertension (HT), arrhythmia, heart failure (HF), chronic renal disease (CRD), dementia, cerebrovascular disease (CVD), and malnutrition. Polypharmacy, frailty, and fall risk were significantly more common in the PS group compared to the normal group, and the median CALLY index was significantly lower in the PS group compared with the normal group [2.2 (1.2–3.1) vs. 2.4 (1.5–3.5), p <0.001].

The study population was further categorized based on their SARC-F assessment results into high (≥4) and normal (<4) risk groups. The sociodemographic features and comorbidities of the sample are summarized in Table 3 and CGA parameters in Table 4. The median age of the high SARC-F group was 77 (72–82) years, while the normal group was 72 (68–77) years (p <0.001). Female sex, BMI, and presence of HT, coronary artery disease (CAD), arrhythmia, HF, CRD, dementia, CVD, malnutrition, living with frailty, polypharmacy, and falls were significantly higher in the high SARC-F group compared to the normal group. Similarly, the median CALLY index was significantly lower in participants with high SARC-F scores than in those with normal SARC-F scores [1.8 (0.9–2.9) vs. 2.4 (1.5–3.4), p <0.001].

The binary logistic regression model revealed the determinants independently associated with sarcopenia risk (Table 5). Higher age, female sex, and living with frailty (CFS ≥4) were significantly associated with increased sarcopenia risk. Lower CALLY index (OR: 0.954, 95% CI: 0.913–0.999; p = 0.047) and MNA-SF scores MNA-SF scores (OR: 0.764, 95%CI: 0.726–0.804, p< 0.001) associated with increased sarcopenia risk. Among the comorbidities, diabetes mellitus (DM) increased the odds of sarcopenia risk by a modest amount, whereas HT, CAD, and CRD were not significantly associated with increased risk. Binary logistic regression analysis identified factors associated with PS (Table 6). Increasing age was significantly associated with a higher odds of PS, and frailty (CFS ≥ 4) substantially increased the odds. Lower CALLY index and MNA-SF scores were associated with higher odds of PS. OR was 0.964 with a 95%CI of 0.932–0.997 (p = 0.031), while female sex, polypharmacy, DM, HT, CAD, and CRD were not significantly associated with PS in the model.

**Figure 2:**
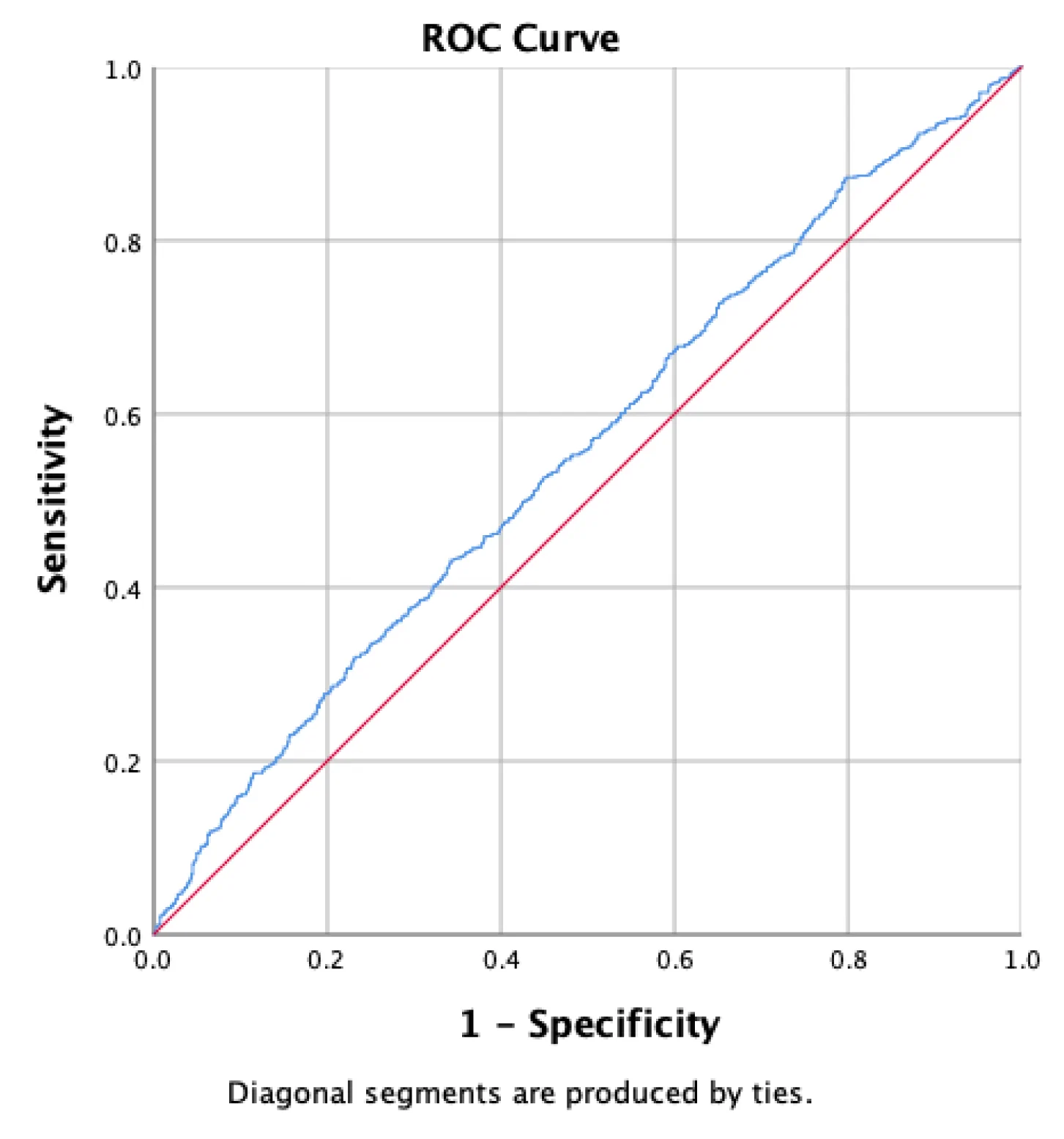
ROC curve of the CALLY index for probable sarcopenia.

**Figure 3:**
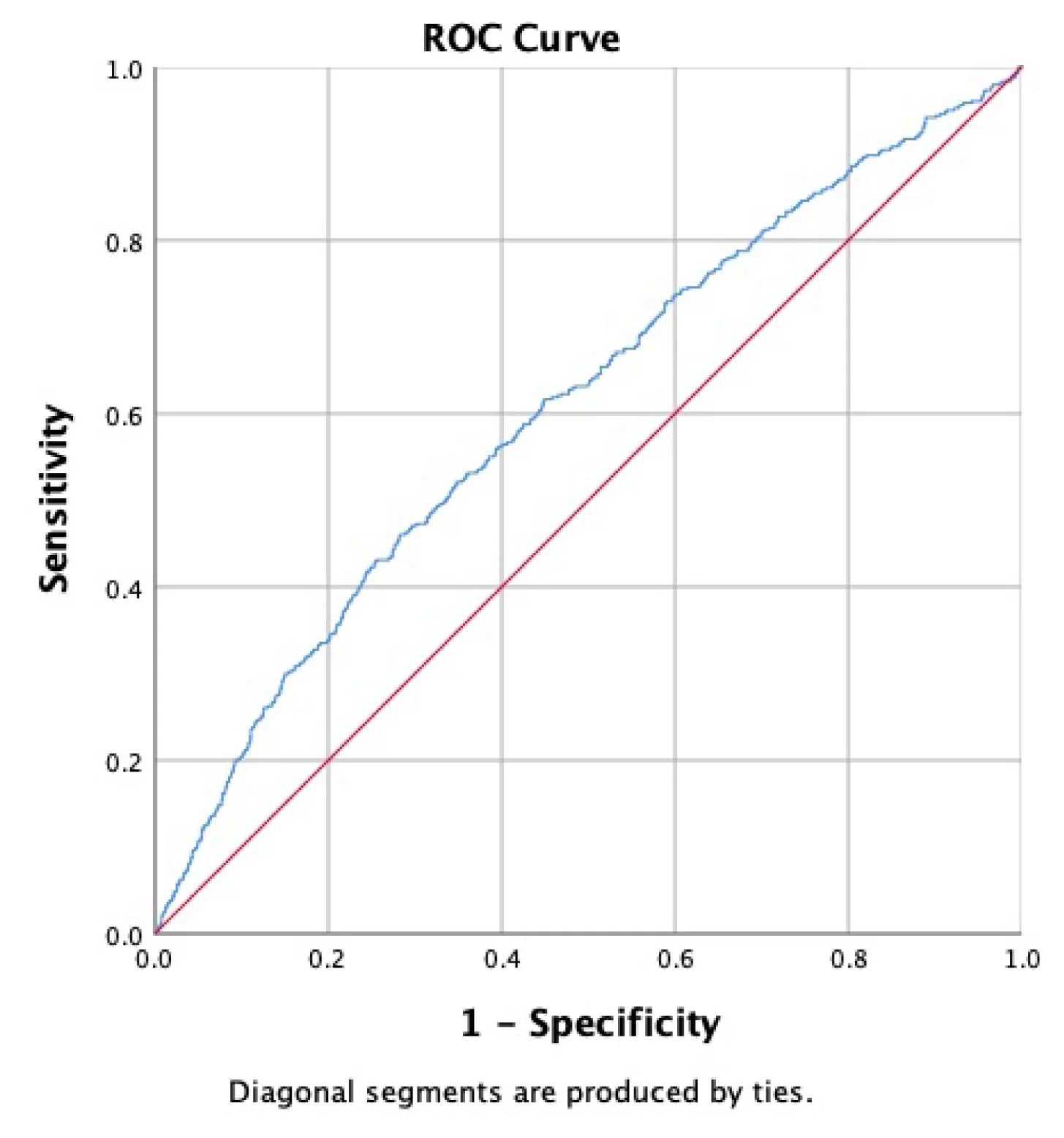
ROC curve of the CALLY index for sarcopenia risk.

ROC curve analysis was performed to evaluate the ability of the CALLY index to discriminate PS, yielding an area under the curve (AUC) of 0.558 (95% CI: 0.533–0.582, p <0.001). ROC analysis for sarcopenia risk, on the other hand, yielded an AUC of 0.609 (95% CI: 0.580–0.638; p <0.001). The optimal cut-off value for the CALLY index for PS was approximately 1.44, with a sensitivity of 31.7% and a specificity of 76.9%. Figure 2 and Figure 3 present the ROC curves of the CALLY index for PS and sarcopenia risk, respectively. Using the observed distribution of positive and negative cases in our cohort, positive predictive value (PPV) and negative predictive value (NPV) were calculated as 54.4% and 56.4%, respectively. Furthermore, for sarcopenia risk, the optimal cut-off value was calculated as approximately 1.91, yielding a sensitivity of 52.1% and a specificity of 65.1%. Based on the observed distribution of sarcopenia risk in our cohort, PPV and NPV were calculated as 31.4% and 81.6%, respectively. Spearman correlation analysis revealed the CALLY index to be negatively associated with SARC-F score (r = − 0.168, p <0.001), TUG (r = −0.168, p <0.001), CST (r = − 0.136, p <0.001), and 4-meter walking time (r = − 0.178, p <0.001), while a positive correlation was observed with HGS (r = 0.105, p <0.001).

## 4. Discussion

This study evaluating the association between the CALLY index and sarcopenia among older adults reveals lower values to be independently and significantly associated with an increased likelihood of sarcopenia. The CALLY index may thus be considered alongside traditional demographic and clinical predictors, although our cross-sectional findings require confirmation through prospective studies.

Sarcopenia is an age-related condition marked by a gradual loss of muscle mass, strength, and functional capacity, and has been linked to such adverse health outcomes as falls, fractures, frailty, increased hospitalization, and a increased risk of mortality [32]. The contribution of chronic inflammatory processes to the onset and progression of sarcopenia has been widely acknowledged in the literature. This persistent low-grade inflammatory state, referred to as inflammaging, is characterized by continuously elevated proinflammatory mediators such as IL-6 and TNF-α [6–8]. These cytokines inhibit muscle protein synthesis while promoting proteolysis, and the inflammatory state produced not only increases muscle breakdown but also impairs the regenerative capacity of satellite cells. A low lymphocyte count indicates both malnutrition and impaired immunity, which further weakens muscle repair mechanisms [37]. Disruptions to the T-cell pathways, particularly CD8+ T cell dysfunction, contribute to ongoing muscle inflammation and mitochondrial impairment, thereby accelerating sarcopenic processes [5]. The relationship between inflammation and immune dysfunction offers a biological basis for why composite indices such as the CALLY index, which reflect inflammation, immune status, and nutritional status, could serve as useful markers associated with sarcopenia [22]. Given the limited availability of practical diagnostic tools in clinical practice, there is a pressing need to identify inflammatory biomarkers that reflect the underlying mechanisms of sarcopenia. Such a strategy may support earlier and easier detection.

Albumin is a liver-derived protein with a long half-life, and it is commonly used in the assessment of an individual’s nutritional status [37–39]. Since malnutrition is one of the common underlying causes of sarcopenia, the inclusion of albumin in this formulation is of particular importance [38]. It should be acknowledged, however, that albumin levels are influenced by inflammatory states as they function as a negative acute-phase reactant, which can lead to decreased serum concentrations independently of malnutrition. Patients with active infections were excluded from the present study, as serum albumin levels within the CALLY index could be influenced by infectious conditions. This approach allowed us to demonstrate the association between the CALLY index and sarcopenia, independent of infection. Previous epidemiological studies have revealed low serum albumin concentrations to be associated with an increased risk of sarcopenia and greater losses of muscle mass over time, highlighting their relevance in both nutritional and muscle health assessments [38, 39].

CRP is increasingly recognized as an acute-phase inflammatory marker with an important role in host responses to infection and inflammation, including apoptosis, complement activation, phagocytosis, and nitric oxide release, and the production of cytokines such as TNF-α [40]. Previous studies have consistently demonstrated that elevated CRP and related inflammatory markers are associated with reduced muscle strength and a greater risk of sarcopenia, underscoring the role of chronic inflammation in its pathophysiology [40–43].

Lymphocytes are involved in regulating immune and inflammatory processes, and age-related declines in T-cell number and function further contribute to these mechanisms that play a role in the development of sarcopenia. Declines in peripheral subsets such as CD3^+^, CD4^+^, and CD8^+^ cells, have been associated with disease progression, reflecting immunosenescence driven by reduced naive T-cell output from thymic involution and the accumulation of dysfunctional CD28- senescent T cells [44]. In addition, diminished B-cell production in the bone marrow and a gradual decline in circulating CD19^+^ B lymphocytes have been reported in older adults in parallel with the development of sarcopenia [45]. Reduced lymphocyte counts may therefore reflect impaired immune-nutritional status, which has been increasingly linked to muscle loss and functional decline in older adults. Consistent with this, lower lymphocyte counts are significantly associated with sarcopenia, supporting the role of immune-nutritional mechanisms in its pathophysiology [46].

The CALLY index was originally introduced as inflammatory marker in a study of cancer patients [47]. More recently, it has been applied not only to malignancies, but also to a range of other diseases and geriatric syndromes, and the accumulating evidence continues to expand the literature [23, 48]. Recent studies have reported associations between lower CALLY levels and an increased risk of early-onset mild cognitive impairment in DM patients, increased mortality in older adults with dysphagia, and poorer prognosis in CAD patients following percutaneous coronary interventions. The index has also been proposed as a prognostic tool in patients with cirrhosis, reflecting both inflammatory and nutritional status [49–53]. In a recent study of hospitalized patients predominantly aged 45–60 years, lower CALLY index levels were associated with an increased risk of sarcopenia [23]. In another study, CALLY levels were able to predict overall survival in sarcopenic cancer patients [21].

It is important to note that no universally accepted reference range for the CALLY index has been established for geriatric populations. In the present study, the median CALLY values were 2.4 (IQR: 1.5–3.5) in the normal group and 2.2 (IQR: 1.2–3.1) in the PS group. Although this difference of 0.2 units was statistically significant in our large sample, its clinical meaningfulness warrants careful interpretation. In the oncology literature, CALLY values <1.0 have been associated with poor prognosis [17, 19], whereas our outpatient population demonstrated generally higher values, likely reflecting a lower inflammatory burden compared with hospitalized or cancer populations. The relatively narrow distribution of CALLY values in our outpatient cohort may partly explain the modest discriminatory ability observed in the ROC analysis. Although the associations between the CALLY index and sarcopenia-related outcomes were statistically significant, the observed effect sizes were relatively modest. The OR values were close to 1, suggesting that changes in the CALLY index are associated with only modest changes in the likelihood of PS or sarcopenia risk. Therefore, while the index may reflect underlying inflammatory and nutritional status, its clinical utility as a standalone marker appears limited. These findings suggest that the CALLY index may be more informative when interpreted alongside other clinical, functional, and nutritional assessments rather than when used as an isolated indicator.

Our evaluation of the association between CALLY levels, PS, and sarcopenia risk revealed lower CALLY levels to be significantly associated with higher PS. All participants were recruited from an outpatient clinic to avoid the potential confounding effects of hospitalization, which can accelerate the development of sarcopenia. Furthermore, by restricting the age range to ≥65 years, results specific to the geriatric population were ensured. A more comprehensive investigation is warranted to further evaluate the prognostic role of the CALLY index in predicting mortality among patients with PS.

There are some limitations to our study, one being the absence of a validated cut-off value for the CALLY index and the relatively low AUC observed in the ROC analysis, which limited its discriminative ability. Although the ROC analysis was statistically significant, we were unable to identify an optimal CALLY threshold for discriminating sarcopenia based on sensitivity and specificity. This may be attributable to the relatively narrower reference ranges of CALLY levels in the outpatient population compared with those reported in the literature. Importantly, the AUC values obtained in this study (0.558 for PS and 0.609 for sarcopenia risk) indicate poor-to-weak discriminative performance for the CALLY index as a standalone marker. An AUC of 0.558 is only marginally above the chance level of 0.50, suggesting limited clinical utility for individual patient classification. Notably, other inflammation-based indices such as the neutrophil-to-lymphocyte ratio (NLR), platelet-to-lymphocyte ratio (PLR), and systemic immune-inflammation index (SII) have also demonstrated similarly modest discriminative ability for sarcopenia in outpatient geriatric settings (AUC range: 0.55–0.65) [10–12]. These findings suggest that the CALLY index, while statistically associated with sarcopenia status, should not be interpreted as a diagnostic tool in isolation, but rather as a single component within a broader clinical assessment framework. Another limitation was that a definitive diagnosis of sarcopenia could not be established because muscle mass was not assessed using muscle ultrasound or dual-energy X-ray absorptiometry (DXA). Therefore, participants were classified as having PS according to the EWGSOP2 criteria. These indicators are cost-effective and widely accessible in routine practice, serving as a simpler, more practical approach to identifying PS compared with current, complex diagnostic methods. Another limitation was the potential confounding effect of chronic diseases that could increase systemic inflammatory burden. Conditions such as DM, CKD, metabolic disorders, nutritional deficiencies, heart failure, obesity, and metabolic syndrome can influence both the CALLY index and sarcopenia risk, given the index’s reliance on CRP and albumin—markers of inflammation, nutritional status, and comorbid disease burden. To address this, we adjusted for the most prevalent comorbidities in our cohort (HT, DM, CAD, and CKD), in line with previous studies [21, 23], and constructed multivariable logistic regression models incorporating variables identified as significant in univariate analyses, as well as those with established clinical relevance. Multicollinearity was assessed prior to model construction, and collinear variables were not included in the same model. Thus, only variables that were significant in the univariate analyses and without problematic multicollinearity were retained in the final regression models. Furthermore, variables were added to regression models based on clinical relevance and evidence from previous studies identifying the factors associated with sarcopenia and related geriatric outcomes. Another important point that should be considered when interpreting our findings is that the outcome of this study reflect PS rather than confirmed sarcopenia according to the EWGSOP2 criteria. As muscle mass measurements were not available in this study, the participants were classified based on muscle strength assessments. Although muscle strength is recommended as the primary indicator of PS in clinical practice, the lack of a muscle mass evaluation in the study prevents confirmation of sarcopenia. Our findings should thus be interpreted within the context of PS, and future studies incorporating direct muscle mass measurements are needed to further clarify the relationship between the CALLY index and confirmed sarcopenia.

Furthermore, this study was conducted at a single university hospital geriatrics outpatient clinic, which may introduce referral bias, as patients seen in such settings tend to be more complex and have a higher comorbidity burden than the general community-dwelling older adult population. Our findings may therefore not be directly generalizable to community-dwelling older adults or primary care settings.

Finally, the reliance of the study on complete case analysis raises the possibility of selection bias in the study, particularly if the participants with missing data systematically differed from those included in the analysis; nevertheless, the relatively large sample size (n = 2042) may have alleviated this potential limitation to some extent.

Among the main strengths of the study are the exclusion of patients with acute or chronic infections, malignancies, and rheumatological diseases. Recognizing that the parameters included in the CALLY index are readily influenced by infection and malignancy, the applied approach sought to provide a clearer assessment of the association between PS and CALLY levels, independent of infection-related confounding. Another strength of our study is its specific focus on patients aged ≥65 years, providing a clearer and more precise depiction of a defined older adult population, unlike previous studies that included a wide age range that were more susceptible to age-related effects.

Future multicenter studies involving diverse clinical settings are needed to validate the findings of our study. Further prospective research is required to determine whether a causal relationship exists between the CALLY index and PS. As this topic has been addressed in only one previous study, our findings provide a valuable contribution to this emerging field [23].

When considering the role of inflammation in sarcopenia, it is evident that practical, accessible laboratory markers are particularly valuable for clinical diagnosis. In this context, CALLY levels can be considered a simple yet informative measure that can aid risk stratification. Although our findings highlight its potential clinical utility, further large-scale, longitudinal studies are warranted to validate its prognostic or predictive value and clarify its role in the routine assessment of sarcopenia.

## 5. Conclusion

Older adults with PS exhibited lower CALLY index values in the present study, suggesting an association between systemic inflammation, nutritional status, and PS. While these findings support the use of the CALLY index as a supplementary marker associated with PS, the cross-sectional nature of this study precludes causal inference, and future longitudinal studies are needed to confirm these associations. Given its limited discriminative ability as a standalone marker, the CALLY index may hold clinical value as one component of a comprehensive geriatric assessment framework rather than as an independent diagnostic tool.

## Figures and Tables

**Table 1:** Baseline characteristic features of the study population according to sarcopenia status.

	Normal (n = 1092)	Probable sarcopenia (n = 950)	p
**Age, median (Q1**–**Q3)**	71 (68–75)	75 (71–80)	**<0.001**
**Sex, female, n (%)**	707 (64.8)	628 (66.2)	0.516
**Marital Status, married, n (%)**	724 (66.4)	522 (55.0)	**<0.001**
**Smoking History, Absent, n (%)**	731 (69.0)	676 (74.0)	**0.039**
**Alcohol history, Absent, n (%)**	947 (90.0)	847 (93.0)	**<0.001**
**BMI, median (Q1**–**Q3)**	28.7 (25.6–31.9)	28.9 (25.3–32.5)	0.491
**CALLY index, median (Q1**–**Q3)**	2.4 (1.5–3.5)	2.2 (1.2–3.1)	**<0.001**
**Diabetes Mellitus, n (%)**	483 (44.2)	401 (42.3)	0.369
**Hypertension, n (%)**	782 (71.6)	733 (77.4)	**0.003**
**Coronary arterial disease, n (%)**	299 (27.3)	297 (31.4)	**0.049**
**Arrhythmia, n (%)**	66 (6.0)	112 (11.8)	**<0.001**
**Heart Failure, n (%)**	30 (2.7)	79 (8.4)	**<0.001**
**Chronic renal disease, n (%)**	51 (4.6)	74 (7.8)	**0.003**
**Depression, n (%)**	187 (18.5)	247 (27.8)	**<0.001**
**Dementia, n (%)**	53 (4.8)	119 (12.6)	**<0.001**
**Cerebrovascular disease, n (%)**	65 (5.9)	91 (9.6)	**0.002**
**Malnutrition, n (%)**	207 (19.0)	344 (36.2)	**<0.001**
**COPD, n (%)**	65 (5.9)	76 (8.0)	0.068

Significant values are presented in bold.

COPD: chronic obstructive pulmonary disease.

**Table 2:** Comprehensive geriatric assessment outcomes of the study population according to sarcopenia status.

	Normal (n = 1092)	Probable sarcopenia (n = 950)	p
**ADL score, median (Q1**–**Q3)**	6 (6–6)	6 (5–6)	**<0.001**
**IADL score, median (Q1**–**Q3)**	8 (8–8)	7 (5–8)	**<0.001**
**Living with Frailty (CFS ≥ 4), n(%)**	438 (40.1)	680 (71.6)	**<0.001**
**CFS score, median (Q1**–**Q3)**	3 (3–4)	4 (3–5)	**<0.001**
**MNA-SF score, median (Q1**–**Q3)**	14 (12–14)	12 (10–14)	**<0.001**
**4-meter walking time (seconds), median (Q1**–**Q3)**	3.8 (3.2–4.5)	5.0 (4.0–6.7)	**<0.001**
**TUG (seconds), median (Q1**–**Q3)**	8.9 (7.3–10.8)	12.2 (9.7–16.1)	**<0.001**
**CST (seconds), median (Q1**–**Q3)**	11.7 (10.0–13.3)	16.8 (14.4–20.0)	**<0.001**
**YGDS score, median (Q1**–**Q3)**	1 (0–4)	2 (0–5)	**<0.001**
**SMMSE score, median (Q1**–**Q3)**	28 (26–30)	26 (23–28)	**<0.001**
**Number of drugs, median (Q1**–**Q3)**	5 (3–8)	7 (4–9)	**<0.001**
**Polypharmacy, n (%)**	584 (62.1)	638 (72.4)	**<0.001**
**Fallers, n (%)**	317 (29.3)	378 (40.2)	**<0.001**
**Osteoporosis, n (%)**	365 (34.6)	385 (42.6)	**<0.001**

Significant values are presented in bold. ADL; activities of daily living, BMI: body mass index, CFS: clinical frailty scale, CST: chair stand test; COPD: chronic obstructive pulmonary disease; IADL: instrumental activities of daily living; MNA-SF: mini-nutritional assessment short-form; SMMSE: standardized mini-mental state examination; TUG: timed up and go, YGDS: Yesevage geriatric depression scale.

**Table 3:** Baseline characteristic features of the study population according to SARC-F status.

	Normal SARC-F (n = 1562)	High SARC-F (n = 480)	p
**Age, median (Q1**–**Q3)**	72 (68–77)	77 (72–82)	**<0.001**
**Sex, female, n (%)**	955 (61.2)	380 (79.3)	**<0.001**
**Marital Status, married, n (%)**	1019 (65.3)	227 (47.3)	**<0.001**
**Smoking History, Absent, n (%)**	1037 (68.6)	370 (80.4)	**<0.001**
**Alcohol history, Absent, n (%)**	1355 (89.9)	439 (96.3)	**<0.001**
**BMI, median (Q1**–**Q3)**	28.6 (25.5–31.6)	29.5 (25.6–34.1)	**0.001**
**CALLY index, median (Q1**–**Q3)**	2.4 (1.5–3.4)	1.8 (0.9–2.9)	**<0.001**
**Diabetes Mellitus, n (%)**	659 (42.2)	225 (46.9)	0.072
**Hypertension, n (%)**	1131 (72.6)	384 (80.0)	**0.001**
**Coronary arterial disease, n (%)**	436 (27.9)	160 (33.5)	**0.020**
**Arrhythmia, n (%)**	107 (6.9)	71 (14.9)	**<0.001**
**Heart Failure, n (%)**	54 (3.5)	55 (11.5)	**<0.001**
**Chronic renal disease, n (%)**	84 (5.4)	41 (8.6)	**0.011**
**Depression, n (%)**	368 (23.6)	210 (44.1)	**<0.001**
**Dementia, n (%)**	93 (6.0)	79 (16.6)	**<0.001**
**Cerebrovascular disease, n (%)**	90 (5.8)	66 (13.8)	**<0.001**
**Malnutrition, n (%)**	300 (19.2)	251 (52.3)	**<0.001**
**COPD, n (%)**	95 (6.1)	46 (9.6)	**0.007**

Significant values are presented in bold.

**Table 4:** Comprehensive geriatric assessment outcomes of the study population according to SARC-F status.

	Normal SARC-F (n = 1562)	High SARC-F (n = 480)	p
**ADL score, median (Q1**–**Q3)**	6 (6–6)	5 (4–6)	**<0.001**
**IADL score, median (Q1**–**Q3)**	8 (8–8)	5 (2–8)	**<0.001**
**Living with Frailty (CFS ≥ 4), n(%)**	684 (43.8)	434 (90.4)	**<0.001**
**CFS score, median (Q1**–**Q3)**	3 (3–4)	5 (4–6)	**<0.001**
**MNA-SF score, median (Q1**–**Q3)**	14 (12–14)	11 (9–13)	**<0.001**
**4-meter walking time (seconds), median (Q1**–**Q3)**	4.0 (3.4–5.0)	6.4 (4.9–8.8)	**<0.001**
**TUG (seconds), median (Q1**–**Q3)**	9.5 (7.8–11.9)	15.1 (11.5–20.9)	**<0.001**
**Chair stand test, median (Q1**–**Q3)**	13.0 (10.8–15.5)	17.9 (14.1–22.8)	**<0.001**
**YGDS score, median (Q1**–**Q3)**	1 (0–3)	4 (2–7)	**<0.001**
**SMMSE score, median (Q1**–**Q3)**	28 (25–29)	25 (20–27)	**<0.001**
**Number of drugs, median (Q1**–**Q3)**	6 (4–8)	8 (5–10)	**<0.001**
**Polypharmacy, n (%)**	877 (63.3)	345 (79.1)	**<0.001**
**Fallers, n (%)**	430 (27.8)	265 (55.8)	**<0.001**
**Osteoporosis, n (%)**	527 (35.3)	223 (48.4)	**<0.001**

Significant values are presented in bold. ADL; activities of daily living, BMI: body mass index, CFS: clinical frailty scale, CST: chair stand test; COPD: chronic obstructive pulmonary disease; IADL: instrumental activities of daily living; MNA-SF: mini-nutritional assessment short-form; SMMSE: standardized mini-mental state examination; TUG: timed up and go, YGDS: Yesevage geriatric depression scale.

**Table 5:** Binary logistic regression analysis of parameters related to probable sarcopenia risk (according to SARC-F ≥4 ).

Sarcopenia Risk	Exp(B)	95% Confidence Interval	p
**CALLY index**	0.955	0.913–0.999	**0.047**
**Age, years**	1.068	1.047–1.089	**<0.001**
**Sex, female**	2.379	1.783–3.176	**<0.001**
**Living with Frailty (CFS ≥ 4)**	6.032	4.286–8.489	**<0.001**
**MNA-SF score**	0.764	0.726–0.804	**<0.001**
**Diabetes Mellitus**	1.397	1.082–1.802	**0.010**
**Hypertension**	0.990	0.725–1.353	0.952
**Coronary Artery Disease**	1.259	0.964–1.645	0.091
**Chronic Kidney Disease**	1.047	0.650–1.687	0.851

Significant values are presented in bold. All variables were included in the regression model. CFS: clinical frailty scale; MNA-SF: mini-nutritional assessment short-form.

**Table 6:** Binary logistic regression analysis of parameters related to probable sarcopenia risk (according to EWGSOP-2 criteria).

Probable Sarcopenia	Exp(B)	95% Confidence Interval	p
**CALLY index**	0.964	0.932–0.997	**0.031**
**Age, years**	1.064	1.045–1.083	**<0.001**
**Sex, female**	0.872	0.700–1.086	0.222
**Living with Frailty (CFS ≥ 4)**	2.690	2.148–3.370	**<0.001**
**MNA-SF score**	0.923	0.880–0.968	**0.001**
**Diabetes Mellitus**	0.903	0.728–1.120	0.353
**Hypertension**	1.128	0.880–1.445	0.341
**Coronary Artery Disease**	0.946	0.750–1.194	0.642
**Chronic Kidney Disease**	1.332	0.862–2.058	0.196
**Polypharmacy**	1.183	0.932–1.501	0.166

Significant values are presented in bold. All variables were included in the regression model. CFS: clinical frailty scale; MNA-SF: mini-nutritional assessment short-form.

## Data Availability

The data underlying this article can be obtained from the corresponding author upon reasonable request.

## References

[1] Franceschi C Garagnani P Parini P Giuliani C Santoro A Inflammaging: a new immune–metabolic viewpoint for age-related diseases Nature Reviews Endocrinology 2018 14 10 576 590 10.1038/s41574-018-0059-4 30046148

[2] Ferrucci L Fabbri E Inflammageing: chronic inflammation in ageing, cardiovascular disease, and frailty Nature Reviews Cardiology 2018 15 9 505 522 10.1038/s41569-018-0064-2 30065258 PMC6146930

[3] Sayer AA Cruz-Jentoft A Sarcopenia definition, diagnosis and treatment: consensus is growing Age and Ageing 2022 51 10 afac220 10.1093/ageing/afac220 36273495 PMC9588427

[4] Petermann-Rocha F Balntzi V Gray SR Lara J Ho FK Global prevalence of sarcopenia and severe sarcopenia: a systematic review and meta-analysis Journal of Cachexia, Sarcopenia and Muscle 2022 13 1 86 99 10.1002/jcsm.12783 34816624 PMC8818604

[5] Zhang N Zhai L Wong RMY Cui C Law S-W Harnessing immunomodulation to combat sarcopenia: current insights and possible approaches Immunity and Ageing 2024 21 1 55 39103919 10.1186/s12979-024-00458-9 PMC11299351

[6] Wang T Searching for the link between inflammaging and sarcopenia Ageing Research Reviews 2022 77 101611 10.1016/j.arr.2022.101611 35307560

[7] Xue J Han X Zheng Y Zhang Q Kong L Effectiveness of resistance training in modulating inflammatory biomarkers among Asian patients with sarcopenia: a systematic review and meta-analysis of randomized controlled trials Frontiers in Immunology 2024 15 1385902 10.3389/fimmu.2024.1385902 38863698 PMC11165069

[8] Antuña E Cachán-Vega C Bermejo-Millo JC Potes Y Caballero B Inflammaging: implications in sarcopenia International Journal of Molecular Sciences 2022 23 23 15039 10.3390/ijms232315039 36499366 PMC9740553

[9] Lucius K Novel and emerging markers of chronic or low-grade inflammation Integrative and Complementary Therapies 2023 29 3 130 142

[10] Öztürk ZA Kul S Türkbeyler İH Sayıner ZA Abiyev A Is increased neutrophil-lymphocyte ratio remarking the inflammation in sarcopenia? Experimental Gerontology 2018 110 223 229 10.1016/j.exger.2018.06.013 29928932

[11] Cataltepe E Ceker E Fadiloglu A Gungor F Karakurt N Association between the systemic immune-inflammation index and sarcopenia in older adults: a cross-sectional study BMC Geriatrics 2025 25 1 28 10.1186/s12877-025-05686-2 39806294 PMC11727228

[12] Xie S Wu Q Association between the systemic immune-inflammation index and sarcopenia: a systematic review and meta-analysis Journal of Orthopaedic Surgery and Research 2024 19 1 314 10.1186/s13018-024-04808-7 38802828 PMC11131329

[13] Liu J Zhang F Associations between sarcopenia, inflammatory markers, and all-cause mortality in older adults: mediation analyses in a large U.S. NHANES sample Frontiers in Medicine 2025 12 1515839 10.3389/fmed.2025.1515839 40708651 PMC12287101

[14] Liaw FY Huang CF Chen WL Wu LW Peng TC Higher platelet-to-lymphocyte ratio increased the risk of sarcopenia in community-dwelling older adults Scientific Reports 2017 7 1 16609 29192175 10.1038/s41598-017-16924-y PMC5709494

[15] Schaap LA Pluijm SM Deeg DJ Harris TB Kritchevsky SB Higher inflammatory marker levels in older persons: associations with 5-year change in muscle mass and strength Journals of Gerontology Series A: Biomedical Sciences and Medical Sciences 2009 64 11 1183 1189 10.1093/gerona/glp097 PMC2759573 19622801

[16] Xu Z Tang J Xin C Jin Y Zhang H Associations of C-reactive protein–albumin–lymphocyte (CALLY) index with cardiorenal syndrome: insights from a population-based study Heliyon 2024 10 17 e37197 10.1016/j.heliyon.2024.e37197 39296012 PMC11408039

[17] Iida H Tani M Komeda K Nomi T Matsushima H Superiority of CRP-albumin-lymphocyte index (CALLY index) as a non-invasive prognostic biomarker after hepatectomy for hepatocellular carcinoma HPB (Oxford) 2022 24 1 101 115 10.1016/j.hpb.2021.05.013 34244053

[18] Özdemir S Özkan A The importance of the CALLY index as a non-invasive prognostic biomarker in SARS-CoV-2 infected patients Medical Science and Discovery 2023 10 7 443 448

[19] Zhang H Shi J Xie H Liu X Ruan G Superiority of CRP-albumin-lymphocyte index as a prognostic biomarker for patients with gastric cancer Nutrition 2023 116 112191 10.1016/j.nut.2023.112191 37716090

[20] Lang S-Q Kong J-J Li G-B Liu J Prognostic value of CRP–albumin–lymphocyte index in patients with intrahepatic cholangiocarcinoma after radical resection Frontiers in Medicine 2025 12 1543665 10.3389/fmed.2025.1543665 40115790 PMC11922830

[21] Wang S Liu C Zheng X Chen Y Liu X Prognostic value of the C-reactive protein–albumin–lymphocyte index in cancer patients with sarcopenia Nutrition 2025 140 112893 10.1016/j.nut.2025.112893 40834469

[22] Oshita K Kobayashi T Honmyo N Shimizu S Kuroda S Prognostic impact of C-reactive protein–albumin–lymphocyte index in patients who underwent splenectomy and devascularization for gastric varices caused by portal hypertension Cureus 2025 17 7 e87092 10.7759/cureus.87092 40747208 PMC12312047

[23] Li Y Wei Q Ke X Xu Y Xu B Higher CALLY index levels indicate lower sarcopenia risk among middle-aged and elderly community residents and hospitalized patients Scientific Reports 2024 14 1 24591 10.1038/s41598-024-75164-z 39426987 PMC11490578

[24] Katz S Ford AB Moskowitz RW Jackson BA Jaffe MW Studies of illness in the aged: the index of ADL JAMA 1963 185 12 914 919 10.1001/jama.1963.03060120024016 14044222

[25] Lawton MP Brody EM Assessment of older people: self-maintaining and instrumental activities of daily living The Gerontologist 1969 9 3 179 186 10.1093/geront/9.3_Part_1.179 5349366

[26] Güngen C Ertan T Eker E Yaşar R Engin F Standardize Mini-Mental Test’in Türk toplumunda geçerliği ve güvenilirliği Türk Psikiyatri Dergisi 2002 13 4 273 281 (in Turkish) 12794644

[27] Molloy DW Alemayehu E Roberts R Reliability of a standardized mini-mental state examination compared with the traditional version American Journal of Psychiatry 1991 148 1 102 105 10.1176/ajp.148.1.102 1984692

[28] Yesavage JA Brink TL Rose TL Lum O Huang V Development and validation of a geriatric depression screening scale: a preliminary report Journal of Psychiatric Research 1982 17 1 37 49 10.1016/0022-3956(82)90033-4 7183759

[29] Guigoz Y Lauque S Vellas BJ Identifying the elderly at risk for malnutrition: the Mini Nutritional Assessment Clinics in Geriatric Medicine 2002 18 4 737 757 10.1016/S0749-0690(02)00059-9 12608501

[30] Sarikaya D Halil M Kuyumcu ME Kılıç MK Yesil Y Mini nutritional assessment test long and short form are valid screening tools in Turkish older adults Archives of Gerontology and Geriatrics 2015 61 1 56 60 10.1016/j.archger.2015.04.006 25944059

[31] Özsürekci C Balcı C Kızılarslanoğlu MC Çalışkan H Tuna Doğrul R Identifying frailty via the 9-point Clinical Frailty Scale Acta Clinica Belgica 2020 75 3 200 204 30919742 10.1080/17843286.2019.1597457

[32] Rockwood K Song X MacKnight C Bergman H Hogan DB A global clinical measure of fitness and frailty in elderly people CMAJ 2005 173 5 489 495 10.1503/cmaj.050051 16129869 PMC1188185

[33] Cruz-Jentoft AJ Bahat G Bauer J Boirie Y Bruyère O Sarcopenia: revised European consensus on definition and diagnosis Age and Ageing 2019 48 1 16 31 10.1093/ageing/afy169 30312372 PMC6322506

[34] Maggio M Ceda GP Ticinesi A De Vita F Gelmini G Instrumental and non-instrumental evaluation of 4-meter walking speed in older individuals PLOS One 2016 11 4 e0153583 10.1371/journal.pone.0153583 27077744 PMC4831727

[35] Malmstrom TK Morley JE SARC-F: a simple questionnaire to rapidly diagnose sarcopenia Journal of the American Medical Directors Association 2013 14 8 531 532 10.1016/j.jamda.2013.05.018 23810110

[36] Bahat G Yilmaz O Kılıç C Oren M Karan M Performance of SARC-F in regard to sarcopenia definitions, muscle mass and functional measures The Journal of Nutrition, Health and Aging 2018 22 8 898 903 10.1007/s12603-018-1067-8 PMC12876385 30272090

[37] Keller U Nutritional laboratory markers in malnutrition Journal of Clinical Medicine 2019 8 6 775 10.3390/jcm8060775 31159248 PMC6616535

[38] Erdoğan K Kara M Şener FE Durmuş ME Durmuşoğlu B Serum albumin as a biomarker of nutritional status in sarcopenia Journal of Bone and Mineral Metabolism 2025 43 2 108 113 10.1007/s00774-024-01557-9 39516399

[39] Visser M Kritchevsky SB Newman AB Goodpaster BH Tylavsky FA Lower serum albumin concentration and change in muscle mass American Journal of Clinical Nutrition 2005 82 3 531 537 16155264 10.1093/ajcn.82.3.531

[40] Lin S Chen X Cheng Y Huang H Yang F C-reactive protein level as a novel serum biomarker in sarcopenia Mediators of Inflammation 2024 2024 3362336 10.1155/2024/3362336 39502753 PMC11535261

[41] Shokri-Mashhadi N Moradi S Heidari Z Saadat S Association of circulating CRP and hs-CRP with components of sarcopenia Experimental Gerontology 2021 150 111330 10.1016/j.exger.2021.111330 33848566

[42] Karanth SD Washington C Cheng T-YD Zhou D Leeuwenburgh C Inflammation in relation to sarcopenia and sarcopenic obesity among older adults Nutrients 2021 13 11 3957 10.3390/nu13113957 34836213 PMC8621174

[43] Choi BH Shin S Association between serum high-sensitivity C-reactive protein levels and low muscle strength among Korean adults Nutrients 2025 17 16 2698 10.3390/nu13113957 40871726 PMC12388901

[44] Liu Z Wang X Li Y Chen Q Sun H Immunosenescence: molecular mechanisms and diseases Signal Transduct Target Therapy 2023 8 144 10.1038/s41392-023-01451-2 PMC10182360 37179335

[45] Ventura MT Casciaro M Gangemi S Buquicchio R Immunosenescence in aging Clinical and Molecular Allergy 2017 15 1 21 29259496 10.1186/s12948-017-0077-0 PMC5731094

[46] Doi S Yasuda S Miyashita M Nagai M Nakamura K Prognostic relevance of sarcopenia and CD8+ T cells in hepatocellular carcinoma Annals of Gastroenterological Surgery 2025 9 2 359 368 40046516 10.1002/ags3.12875 PMC11877349

[47] Wu B Liu J Shao C Yu D Liao J Integrating inflammation, nutrition, and immunity: the CALLY index as a prognostic tool in digestive system cancers – a systematic review and meta-analysis BMC Cancer 2025 25 1 672 40217204 10.1186/s12885-025-14074-3 PMC11992890

[48] Luo L Li M Xi Y Hu J Hu W C-reactive protein–albumin–lymphocyte index as a feasible nutrition-immunity-inflammation marker Clinical Nutrition ESPEN 2024 63 346 353 10.1016/j.clnesp.2024.04.019 38985666

[49] Lin CY Zhai YJ Wu F An HH Chen T Interaction of underweight, low muscle mass, malnutrition, and inflammation with early-onset mild cognitive impairment in type 2 diabetes Frontiers in Aging Neuroscience 2025 17 1498478 10.3389/fnagi.2025.1498478 40212567 PMC11983464

[50] Han D Wu L Zhou H Xue Y He S Associations of the C-reactive protein–albumin–lymphocyte index with all-cause and cardiovascular mortality BMC Cardiovascular Disorders 2025 25 1 144 40025412 10.1186/s12872-025-04596-w PMC11874105

[51] Pan Y Wu TT Deng CJ Jiang ZH Yang Y Association between the C-reactive protein–albumin–lymphocyte index and adverse clinical outcomes in CAD after PCI Reviews in Cardiovascular Medicine 2024 25 4 111 10.31083/j.rcm2504111 39076545 PMC11264017

[52] Yıldız G Bedel C Zortuk Ö Selvi F Karanci Y CALLY index but not HALP score is associated with mortality in cirrhosis Revista Română de Medicină de Laborator 2025 33 1 29 34 10.2478/rrlm-2025-0005

[53] Shen H Jin T Mei Q Guan H Yan K Association between the C-reactive protein–albumin–lymphocyte index and mortality in elderly patients with dysphagia requiring nutritional support Frontiers in Neurology 2025 16 1650795 10.3389/fneur.2025.1650795 41098380 PMC12518111

